# Endothelium Expression of Bcl-2 Is Essential for Normal and Pathological Ocular Vascularization

**DOI:** 10.1371/journal.pone.0139994

**Published:** 2015-10-07

**Authors:** Ismail S. Zaitoun, Ryan P. Johnson, Nasim Jamali, Reem Almomani, Shoujian Wang, Nader Sheibani, Christine M. Sorenson

**Affiliations:** 1 Department of Ophthalmology and Visual Sciences, University of Wisconsin, Madison, WI, 53705, United States of America; 2 Department of Pediatrics, University of Wisconsin, Madison, WI, 53705, United States of America; 3 McPherson Eye Research Institute, University of Wisconsin, Madison, WI, 53705, United States of America; Indiana University College of Medicine, UNITED STATES

## Abstract

Bcl–2 is an anti-apoptotic protein with important roles in vascular homeostasis and angiogenesis. Mice globally lacking Bcl–2 (Bcl–2 -/-) are small in stature and succumb to renal failure shortly after weaning as a result of renal hypoplasia/cystic dysplasia. We have shown that Bcl–2 -/- mice displayed attenuated retinal vascular development and neovascularization. *In vitro* studies indicated that in addition to modulating apoptosis, Bcl–2 expression also impacts endothelial and epithelial cell adhesion, migration and extracellular matrix production. However, studies delineating the cell autonomous role Bcl–2 expression plays in the endothelium during vascular development, pruning and remodeling, and neovascularization are lacking. Here we generated mice carrying a conditional Bcl–2 allele (Bcl-2^Flox/Flox^) and VE-cadherin-cre (Bcl-2^EC^ mice). Bcl-2^EC^ mice were of normal stature and lifespan and displayed some but not all of the retinal vascular defects previously observed in global Bcl–2 deficient mice. Bcl-2^EC^ mice had decreased numbers of endothelial cells, decreased retinal arteries and premature primary branching of the retinal vasculature, but unlike the global knockout mice, spreading of the retinal superficial vascular layer proceeded normally. Choroidal neovascularization was attenuated in Bcl-2^EC^ mice, although retinal neovascularization accompanying oxygen-induced ischemic retinopathy was not. Thus, Bcl–2 expression in the endothelium plays a significant role during postnatal retinal vascularization, and pathological choroidal but not retinal neovascularization, suggesting vascular bed specific Bcl–2 function in the endothelium.

## Introduction

Apoptosis, or programmed cell death, plays an important role in organ development and maintenance by removing mutated, damaged or unwanted cells in both plant and animal systems [1]. Bcl–2 is the founding member of a family of proteins that influence apoptosis. Family members contain conserved regions denoted as Bcl–2 homology (BH) domains. Anti-apoptotic mammalian Bcl–2 family members share up to four BH domains as well as a transmembrane spanning region at the carboxyl end of the protein [2]. Anti-apoptotic family members include Bcl–2, bcl-X_L_, bcl-w, mcl1 and A1 [3, 4]. Pro-apoptotic members are divided into those that only contain a BH3 domain and those that contain multiple BH domains (typically BH1, BH2 and BH3) [5]. Bim, bid, bad, bik, Puma, bmf, Noxa and Hrk are BH3 only containing pro-apoptotic proteins while, bax and bak are pro-apoptotic proteins that contain multiple BH domains. The regulated expression of this family of proteins during development and their mechanism of action remains a topic of considerable interest.

Although Bcl–2 role in inhibiting apoptosis is well defined, our recent studies which illustrate its influence on the extracellular matrix (ECM) milieu, cell adhesion and migration begin to paint a broader picture of its influence during tissue morphogenesis [6–8]. The ECM not only provides cell structural support but also modulates cell survival, migration, proliferation and differentiation [9], thus changes in the ECM milieu can impact tissue structure and function, including vascularization. Perhaps the ability of a cell to sense its three dimensional location through its interactions with the ECM and neighboring cells impacts vascular development which is essential for tissue integrity and function. Disruption of this delicately balanced microenvironment can also lead to many disease states.

Modulation of Bcl–2 expression plays a central role during angiogenesis. Bcl–2 can act as a pro-angiogenic factor by inducing VEGF and HIF–1 expression, independent of its role in preventing apoptosis [10, 11]. Anti-angiogenic factors can inhibit angiogenesis by down regulation of Bcl–2 expression with subsequent endothelial cell apoptosis [12, 13]. We have previously shown that Bcl–2 family members play a central role in development, remodeling and pruning of the vasculature in multiple organs including retina, kidney and lung [8, 14, 15]. In the retina, global Bcl–2 deficient mice display decreased numbers of retinal endothelial cells and pericytes, reduced numbers of retinal arteries, increased apoptosis and proliferation, delayed progression of the superficial layer of retinal vessels and attenuated ischemia-driven retinal neovascularization [15]. However, the contribution of specific vascular cell types lacking Bcl–2 to this phenotype is not known.

Here we determined the impact of Bcl–2 expression in the endothelium on postnatal retinal vascularization, pathologic retinal neovascularization in oxygen-induced ischemic retinopathy (OIR) and laser-induced choroidal neovascularization (CNV). Mice conditionally lacking Bcl–2 expression in endothelial cells (Bcl-2^EC^ mice) demonstrated increased levels of apoptosis and proliferation but decreased numbers of retinal endothelial cells, retinal arteries, distance to primary branching and pathological choroidal neovascularization. However, the spreading of the retinal vascular superficial layer and ischemia-driven neovascularization in Bcl-2^EC^ mice were similar to their wild-type counterparts. Thus, a balanced expression of pro- and anti-apoptotic Bcl–2 family members in vascular cells is essential to maintain normal vascular function, and their disruption may adversely impact vascularization and pathologic neovascularization in a tissue specific manner.

## Materials and Methods

### Ethics Statement

Experiments were performed in accordance to the Association for Research in Vision and Ophthalmology Statement for the Use of Animals in Ophthalmic and Vision Research and were approved by the Institutional Animal Care and Use Committee of the University of Wisconsin School of Medicine and Public Health.

### Animals

The VE-cadherin-cre (B6.Cg-Tg(Cdh5-cre)7Mlia/J;Jackson Laboratory, Bar Harbor, ME; stock number 006137) and Bcl-2^Conditional^ (bcl2tmlIrt/J;Jackson Laboratory stock number 008882) were maintained at the University of Wisconsin animal facilities according to approved protocols. Genotyping of VE-cadherin-cre mice was accomplished by PCR analysis of genomic DNA extracted from tail biopsies using the following primers 5’-GCGGTCTGGCAGTA AAAACTATC–3’ and 5’-GTGAAACA GCATTGCTGTCACTT–3’. Genotyping of the Bcl-2^Conditional^ mouse line was screened with the following primers: 5’-GCCCACCATCTAAAGAGCAA–3’ and 5’- GCATTT TCCCACCACTGTCT–3’. We bred mice homozygous for the Bcl-2^Conditional^ allele with the VE-cadherin-cre mice to obtain heterozygous mice for the Bcl-2^Conditional^ allele that expressed VE-cadherin-cre. These mice were bred and the progeny screened as described above to obtain mice homozygous for the Bcl-2^Conditional^ allele, also expressing VE-cadherin-cre. We maintained the colony by breeding mice homozygous for the Bcl-2^Conditional^ allele expressing VE-cadherin-cre to mice homozygous for the Bcl-2^Conditional^ allele and genotyping. Mice homozygous for the Bcl-2^Conditional^ allele that express VE-cadherin-cre are referred to as Bcl-2^EC^. C57BL/6J mice are referred to as wild-type. To assess Cre-mediated excision we crossed mice carrying a conditional Tomato allele to VE-cadherin-cre mice. We only observed Tomato expression in retinal endothelial cells (data not shown).

For OIR, 7-day-old (P7) pups with the dam were placed in an airtight incubator and exposed to an atmosphere of 75±0.5% oxygen for 5 days. Incubator temperature was maintained at 23±2°C, and oxygen was continuously monitored with a PROOX model 110 oxygen controller (Reming Bioinstruments Co., Redfield, NY). Mice were then brought to room air for 5 days, and retinal wholemounts prepared [14, 15].

### Trypsin-Digested Retinal Vessel Preparation

Eyes were enucleated from P21 or P42 mice and fixed in 4% paraformaldehyde for at least 24 h. The eyes were bisected equatorially and the entire retina was removed under the dissecting microscope. Retinas were washed overnight in distilled water, and incubated in 3% trypsin (Trypsin 1:250, Difco) prepared in 0.1 M Tris, 0.1 M maleic acid, pH 7.8 containing 0.2 M NaF for approximately 1–1.5 h at 37°C. Following completion of digestion, retinal vessels were flattened by four radial cuts and mounted on glass slides for periodic acid-schiff (PAS) and hematoxylin staining. Nuclear morphology was used to distinguish pericytes from endothelial cells. The nuclei of endothelial cells are oval or elongated and lie within the vessel wall along the axis of the capillary, while pericyte nuclei are small, spherical, stain densely, and generally have a protuberant position on the capillary wall. The stained and intact retinal wholemounts were coded, and subsequent counting was performed masked.

The number of endothelial cells and pericytes was determined by counting respective nuclei per field of view under the microscope at a magnification of x400. Only retinal capillaries were included in the cell count, which was performed in the mid-zone of the retina. We counted the number of endothelial cells and pericytes in four fields of view from the four quadrants of each retina. To evaluate the density of cells in the capillaries, the mean number of endothelial cells or pericytes was recorded in four fields of view from the four quadrants of each retina.

### Visualization of Retina Vasculature and Quantification of Avascular Area

At various times the eyes of mice were enucleated and briefly fixed in 4% paraformaldehyde (10 min on ice). The eyeballs were fixed in 70% methanol for at least 24 h at -20°C. Retinas were dissected in PBS and then washed with PBS three times, 10 min each. Following incubation in a blocking buffer (50% fetal calf serum, 20% normal goat serum in PBS) for 2h, the retinas were incubated with anti-collagen IV (diluted 1:250 in PBS containing 20% fetal calf serum, 20% normal goat serum) at 4°C overnight. In some cases retinas were also stained with anti-α-smooth muscle actin (SMA; Sigma). Retinas were then washed three times with PBS, 10 min each, incubated with secondary antibody Alexa 594 goat-anti-rabbit (Invitrogen; 1:500 dilution prepared in PBS containing 20% FCS, 20% NGS) for 2 hours at RT, washed four times with PBS, 30 min each, and mounted on a slide with PBS/glycerol (2vol/1vol). Retinas were viewed by fluorescence microscopy and images were captured in digital format using a Zeiss microscope (Carl Zeiss, Chester, VA). The central capillary dropout area was quantified, as a percentage of the whole retina area, from the digital images in masked fashion using Axiovision software (Carl Zeiss, Chester, VA). Quantification of vitreous neovascularization was performed as previously described by Stahl et al. [16].

### Laser Induced Choroidal Neovascularization (CNV)

CNV was induced (6 week old, female) by laser photocoagulation-induced rupture of Bruch’s membrane on Day 0. Since female mice were used the data obtained only apply to female mice. Mice were anesthetized with ketamine hydrochloride (80 mg/kg) and xylazine (10 mg/kg) and the pupils were dilated with one drop of tropicamide (1%). Laser photocoagulation (75 μm spot size, 0.1 sec duration, 120 mW) was performed in the 9, 12, and 3 o’clock positions of the posterior pole of each eye with the slit lamp delivery system of an OcuLight GL diode laser (Iridex, Mountain View, CA) and a handheld cover slip as a contact lens to view the retina. After 14 days (ICAM–2) or 3 days (F4/80), the eyes were removed and fixed in 4% paraformaldehyde at 4°C for 2 hours. Following three washes in PBS, the eyes were sectioned at the equator, and the anterior half, the vitreous, and the retina removed. The remaining eye tissue was incubated in blocking buffer (5% fetal calf serum, 20% normal goat serum in PBS) for 1 hour at room temperature, followed by incubation with anti-ICAM–2 (BD Pharmagen;1:500 in PBS containing 20% fetal calf serum, 20% normal goat serum) or F4/80 (eBiosciences) overnight at 4°C. The remaining eye tissue was then washed three times with PBS and incubated with the appropriate secondary antibody. The retinal pigment epithelium-choroid-sclera complex was dissected through five to six relaxing radial incisions and flatmounted on a slide with PBS/glycerol (1vol/1vol). The remaining eye tissue was viewed by fluorescence microscopy and images were captured in digital format using a Zeiss microscope (Zeiss, Chester, VA). Image J software (National Institute of Mental Health, Bethesda, MD; http://rsb.info.nih.gov/ij/) was used to measure the total area (in μm^2^) of CNV associated with each burn.

### BrdU Staining of Wholemount Retinas

The detection of cellular proliferation on the retinal blood vessels was assessed by immunohistochemistry for 5-bromo-2-deoxyuridine (BrdU) incorporation. Mice were injected intraperitoneally with 5-bromo-2-deoxyuridine (BrdU; Sigma, St. Louis, MO) 0.12g per kilogram of body mass dissolved in water. Two hours later the animals were sacrificed, and eyes were removed and fixed immediately in 4% paraformaldehyde for 3 min on ice. Eyes were then transferred to methanol and stored at –20°C for 2 to 72 h. Retinas were dissected in PBS, washed for 30 min in PBS containing 1% Triton X–100 to permeabilize cell membranes, and placed in 2 M HCl at 37°C for 1 h. Each retina was then washed in 0.1M sodium borate for 30 min to neutralize the HCl. Retinas were then washed in PBS containing 1% Triton X–100 for 15 min and incubated with a monoclonal antibody to BrdU (Cat No. 1170376, Roche, Indianapolis, IN; diluted 1:250 in PBS containing 1% bovine serum albumin, BSA) and Isolectin B4-FITC (Vector) at 4°C overnight or at room temperature for 2 h. Following incubation, retinas were washed for 10 min in PBS containing 1% Triton X–100 and incubated with anti-mouse CY5 antibody (Jackson ImmunoResearch Laboratories) diluted 1:500 in PBS containing 1% BSA for 2 h. After a final wash in PBS for 30 min, the retinas were mounted with the ganglion cell layer uppermost in PBS:glycerol (2 vol/1 vol). Retinas were viewed by fluorescence microscopy and images were captured in digital format using a Zeiss microscope (Carl Zeiss, Chester, VA). For quantification, the numbers of BrdU-positive nuclei on the blood vessels were determined per retina.

### Smooth Muscle Actin (SMA) Staining

The eyes were enucleated and fixed for 5 minutes and transferred to methanol until use. The retinas were dissected so that the optic nerve remained. The retinas were then washed three times in PBS and blocked for 1 hour in blocking buffer (50% fetal calf serum (FCS), 20% normal goat serum and 0.1% Triton X–100 in PBS). Anti-SMA-FITC (1:500; Sigma F3777) was diluted in 20% fetal calf serum, 20% goat serum and 0.1% Triton X–100 and incubated with the retinas overnight at 4°C. The retinas were then washed three times in PBS and mounted. The number of arteries and veins per retina were quantified as well as the distance from the center of the optic nerve to primary and secondary branch points.

### Cleaved Caspase 3 Staining

Eyes were enucleated and fixed for 3 minutes in 4% paraformaldehyde and stored in methanol at -20°C overnight. The retinas were dissected and placed in PBS for 30 minutes, fixed in 3% paraformaldehyde for 30 minutes and washed three times in in PBS. The retinas were then transferred to new tubes, rinsed and blocked in 50% blocking buffer (see above) for 24 hours at room temperature. Next the retinas were incubated with anti-cleaved caspase (1:50; Cell Signaling clone D3E9 # 9579) in 2.5% BSA, 0.4% Triton X–100 and 5% blocking buffer for 24 hours at 4°C while rocking, washed 5 times in PBS, one time in 2.5% BSA, 5% blocking buffer and 0.4% Triton X–100 (20 minutes at room temperature) and then rocked in 50% blocking buffer at room temperature for 30 minutes. The retinas were then incubated with the appropriate secondary antibody (1:500; Jackson ImmunoResearch Laboratories) in 2.5% BSA, 5% blocking buffer and 0.4% Triton X–100 rocking for 2 hours at room temperature. The samples were washed three times in PBS for 10 minutes and fixed in 3% paraformaldehyde for 30 minutes at room temperature. The retina were then washed 3 times in PBS, transferred to new tubes and washed once more in PBS. The retinas were then incubated in Isolectin B4-FITC (1:100: Vector Labs) for 90 minutes and washed. The samples were mounted in mounting medium with DAPI (Southern Biotech). For quantification, the numbers of cleaved capase positive cells on the blood vessels were determined per retina.

### Staining Frozen Sections

Mouse eyes were enucleated and embedded in optimal cutting temperature (OCT) compound and 9 μm sections cut. Sections were fixed in cold acetone (48C) on ice for 10 min, washed with PBS, blocked (1% BSA, 0.2% skim milk, and 0.3% Triton X–100 in PBS) at room temperature and incubated with FITC labeled *Bandeiraea simplicifolia* lectin (B4 lectin; Sigma). Retina sections were viewed by fluorescence microscopy and images were captured in digital format using an Evos microscope.

### Statistical Analysis

Statistical differences between groups were evaluated with the student’s unpaired t-test (two-tailed). Mean ± standard error is shown. P<0.05 is considered significant.

## Results

### Decreased Endothelial Cell Numbers in Bcl-2^EC^ Mice

Postnatal vascularization of the mouse retina provides a unique opportunity to study the coordinated regulation of endothelial cells, pericytes and astrocytes during vascular development. We have shown that germline deletion of Bcl–2 adversely affects retinal vascularization and pathological neovascularization [15]. However, the role Bcl–2 expression in endothelial cells plays during these processes is not well understood. To address this question we generated mice homozygous for the Bcl-2^Flox/Flox^ allele which also expressed VE-cadherin-cre referred to as Bcl-2^EC^ mice. The VE-cadherin-cre mice have been well characterized [17] and utilized in numerous studies [18–21]. These mice demonstrate Cre activity in arteries, veins and capillaries. The constitutive nature of this promotor is advantageous for gene deletion in pathological settings which is why it was chosen for the studies shown here [17].

**Fig 1:**
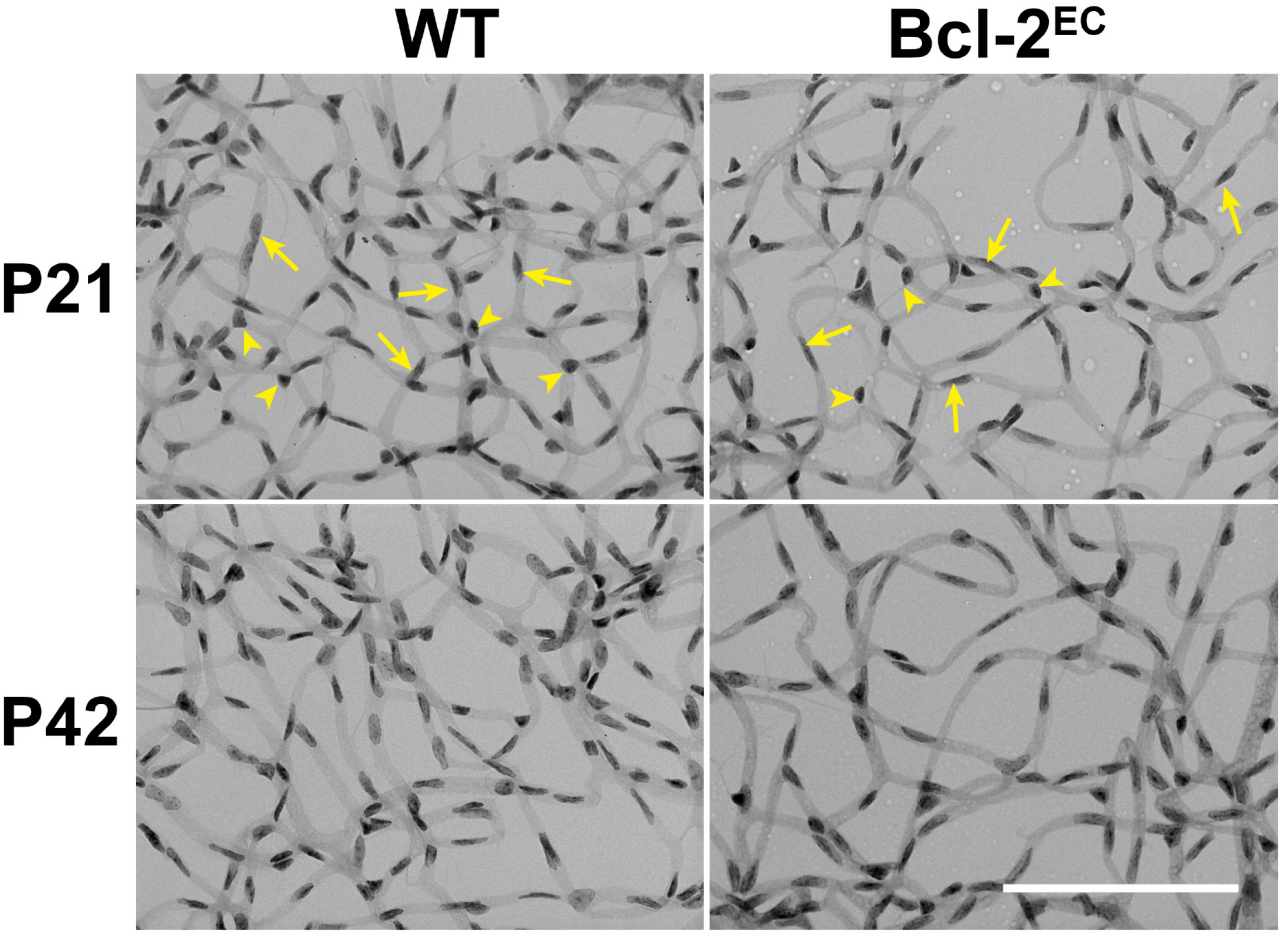
Decreased endothelial cell number in retinas from Bcl-2^EC^ mice. Retinas from P21 and P42 Wild-type (WT) and Bcl-2^EC^ mice were prepared by trypsin digest and HE/PAS staining. Endothelial cells (arrow) and pericytes (arrowhead) were then quantitated per x400 field of view. Scale bar = 100 μm. Experiments were repeated with eyes from >5 mice with similar results. The quantitative assessment of this data is summarized in Table 1.

Although vascularization of the mouse retina is complete by postnatal day 21 (P21), remodeling and pruning of unnecessary vessels occurs until P42 [22]. To assess the impact endothelial Bcl–2 expression has on retinal vascular development, retinal endothelial cell and pericyte numbers were calculated from retinal trypsin digest preparations from P21 and P42 wild-type (WT) and Bcl-2^EC^ mice. On trypsin digest preparations, endothelial cell nuclei appear large, oval, and weakly stained within the vessel wall (arrow), while pericyte nuclei are darkly stained, small, round, and protrude laterally from the vessel wall (arrowhead) (Fig 1). Bcl-2^EC^ mice demonstrated decreased numbers of endothelial cells in trypsin digest preparations compared to their wild-type counterparts (Table 1 and Fig 1). Here we show that retinal endothelial cell numbers in both wild-type and Bcl-2^EC^ mice decreased by P42 suggesting that pruning and remodeling may occur independent of Bcl–2 expression. Although, pericyte numbers were similar in P21 wild-type and Bcl-2^EC^ mice (Table 1), only wild-type mice demonstrated a decrease in pericyte number at P42 due to remodeling and pruning. Perhaps maintaining pericyte numbers during retinal vascular remodeling in Bcl-2^EC^ mice protected the vasculature from even more vascular cell loss during this process.

**Table 1:** The quantitative assessments of retinal vascular cells at different postnatal days.

Mean Number of Cells Per High Power Field (x400) at P21		
** **	Control	Bcl-2^EC^
Pericytes (PC)	32.60 ± 0.9852 (20)	34.64 ± 0.8942 (28)
Endothelial Cell (EC)	184.8 ± 4.325 (20)	169.1 ± 3.027 (28)*
Mean Number of Cells Per High Power Field (x400) at P42		
** **	Control	Bcl-2^EC^
Pericytes (PC)	28.08 ± 0.6913 (24)	35.89 ± 0.7805 (36)*
Endothelial Cell (EC)	164.8 ± 3.515 (24)	136.3 ± 2.953 (36)*

*P<0.01; The number of retinas (mice) is given in parentheses.

### Lack of Endothelial Bcl–2 Expression Decreases Retinal Artery and Vein Numbers

**Fig 2:**
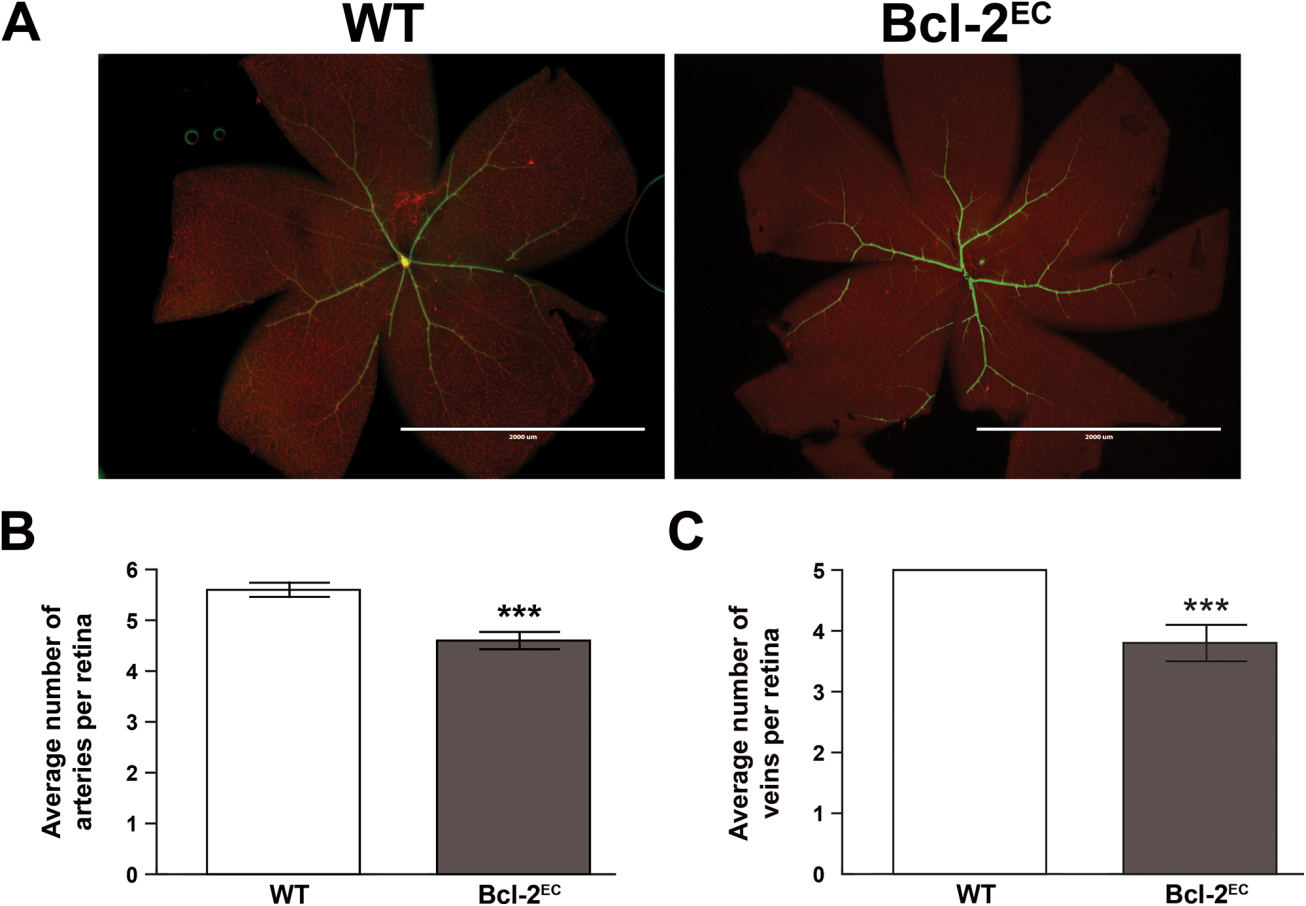
Decreased numbers of retinal arteries in Bcl-2^EC^ mice. Retinas from P21 Wild-type and Bcl-2^EC^ mice were wholemount stained with anti-α-smooth muscle actin (green) and anti-collagen IV (red) to identify major arteries and capillaries, respectively (Panel A). In Panel B the number of arteries and in Panel C the number veins per retina were quantified (***P< 0.001; n = 15). Scale bar in Panel A = 2000 μm.

**Fig 3:**
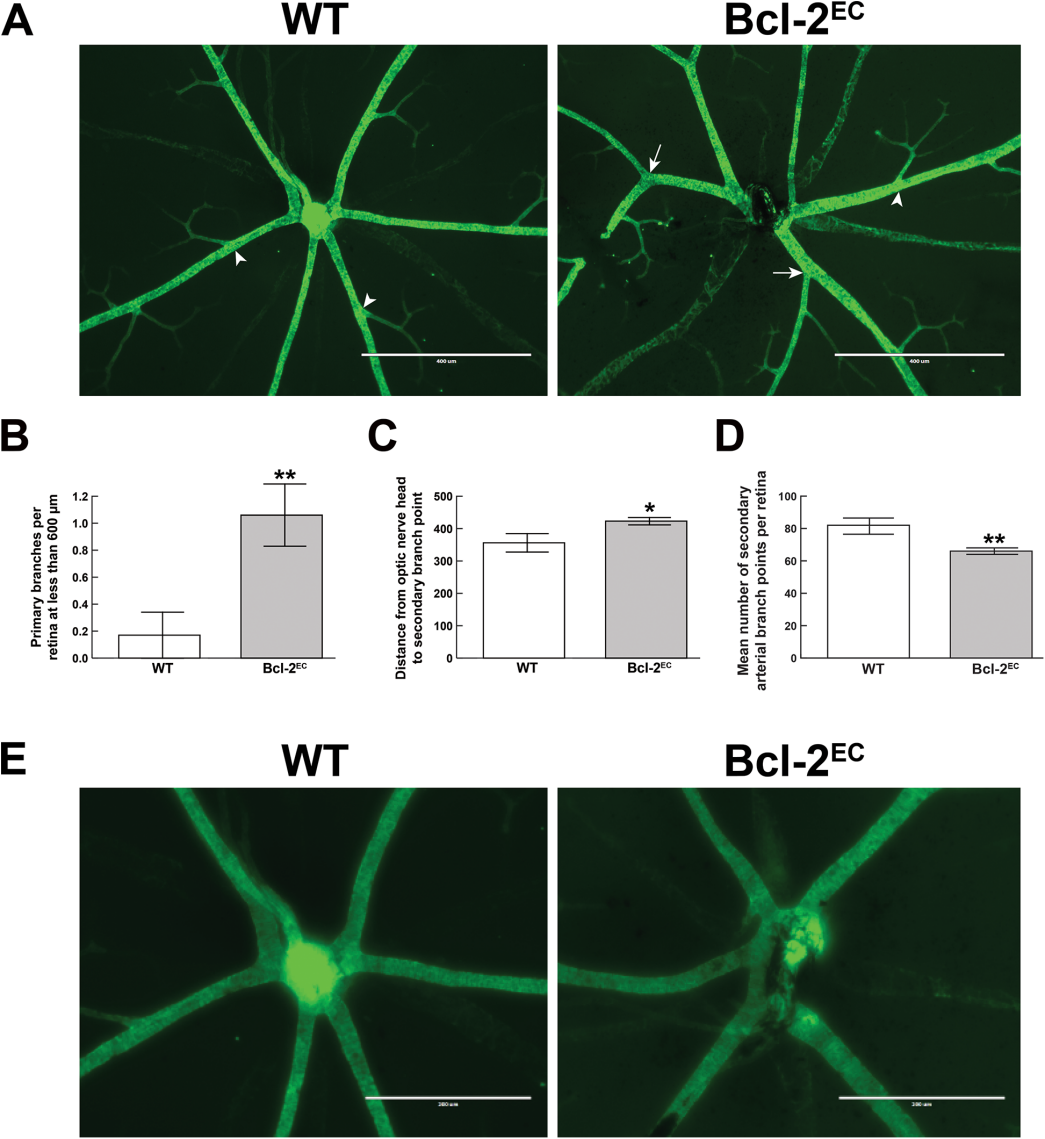
Premature primary branching in Bcl-2^EC^ mice. Retinas from P21 mice were wholemount stained with anti-α-smooth muscle actin, Panel A (scale bar = 400 μm). The distance of primary (arrow) and secondary branches (arrowhead) for the center of the optic nerve were determined as shown in Panels B (**P< 0.001) and C (*P< 0.05) respectively. Panel D demonstrates the numbers of secondary branch points off the retinal arteries. In Panel E, photomicrographs were taken of retinal arteries near the optic nerve (scale bar = 200 μm). Please note symmetrical branching of retinal arteries from wild-type mice. Experiments were repeated with eyes from at least 5 mice with similar results.

The processes involved in determination of retinal artery number are not well understood. We next examined whether Bcl–2 expression in endothelial cells impacted the number of major arteries or veins in the retina. Fig 2A demonstrates retinas from P21 wild-type and Bcl-2^EC^ mice wholemount stained with SMA and collagen IV. Typically in wild-type mice we noted 5–6 retinal arteries (5.57 ± 0.17 arteries; Fig 2B). However, in the absence of endothelial Bcl–2 expression we noted a decreased number of retinal arteries (4.68 ± 0.78 arteries; Fig 2B), which coincided with decreased number of retinal veins (5±0 WT vs. 3.8± 0.3 Bcl-2^EC^; Fig 2C). Primary and secondary branching off of the retinal artery was also aberrant in Bcl-2^EC^ mice. Primary branching occurred prematurely in the absence of endothelial Bcl–2 expression (Fig 3B), while secondary branching was observed at a greater distance from the optic nerve (Fig 3C) and demonstrated fewer secondary arterial branch points (Fig 3D) than in wild-type mice. Next we examined bifurcation of the retinal artery near the optic nerve at higher magnification (Fig 3E). Typically bifurcation of the retinal artery occurred near the optic nerve in wild-type mice. In contrast, in Bcl-2^EC^ mice the branching of the retinal artery was aberrant, appearing as if branching occurs too late after the central retinal artery pierces the optic nerve. Thus, Bcl-2^EC^ mice demonstrated decreased artery numbers and aberrant branching.

### Spreading of the Superficial Layer of Vessels Proceeds Normally in Retinas from Bcl-2^EC^ Mice

**Fig 4:**
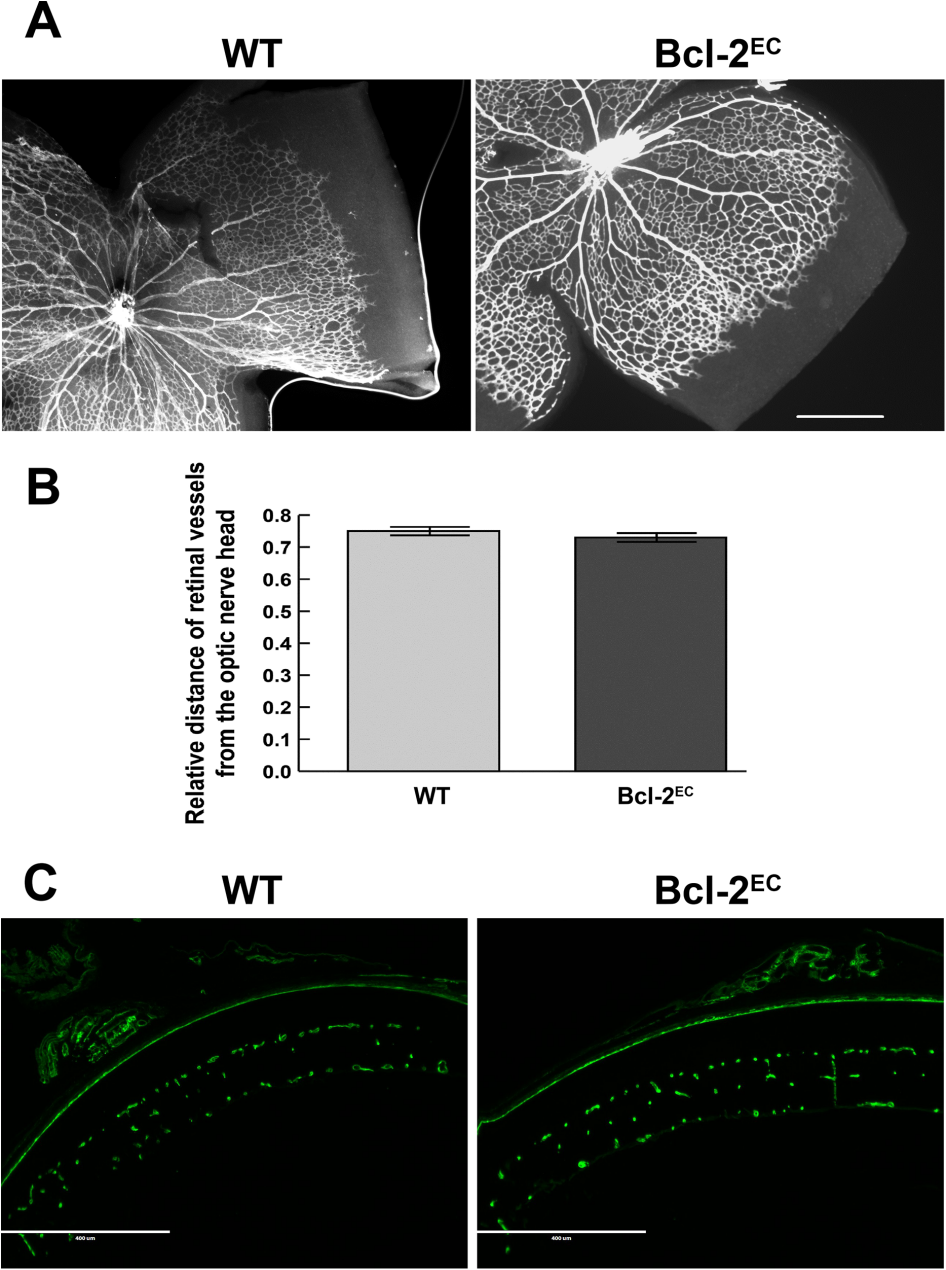
Lack of Bcl–2 expression in endothelial cells does not affect the distance of migrating front from the optic nerve. Collagen IV staining of the retinal wholemount preparations from P5 wild-type and Bcl-2^EC^ mice (Panel A) were quantitatively assessed for the distance retinal vessels spread relative to the radius of the retina at P5 (Panel B). Please note that the spreading of retinal vessels is similar in wild-type and Bcl-2^EC^ mice (P>0.05). In Panel C, frozen sections from P14 wild-type and Bcl-2^EC^ mice were stained with B4 lectin. Please note that the vascular plexus formation occurs in retinas from both wild-type and Bcl-2^EC^ mice. Eyes from at least 5 mice were examined and measurements taken in each quadrant. Scale bar = 500 μm (Panel A) and 400 μm (Panel C).

In the mouse, postnatal progression of retinal vascularization begins at birth. During the first week of life the superficial layer of the retinal vasculature is formed. Our previous studies demonstrated a delay in spreading of the retinal superficial vascular layer in global Bcl–2 deficient mice. Here we addressed whether lack of Bcl–2 expression in endothelial cells influenced spreading of the superficial retinal vascular layer. At P5 the relative distance the retinal vessels had migrated from the optic nerve was similar in retinas from both wild-type and Bcl-2^EC^ mice with approximately 75% of the retina was covered with a superficial layer of vessels (Fig 4A and 4B). To assess whether the vascular plexus formed we stained frozen retinal sections from P14 mice with B4 lectin to visualize the vasculature. Fig 4C demonstrates the formation of the intermediate and deep vascular plexus occurs both in retina from P14 wild-type and Bcl-2^EC^ mice. Thus, formation of the retinal superficial layer and deep vascular plexus does not rely on endothelial Bcl–2 expression.

### Bcl-2^EC^ Mice Demonstrate Increased Apoptosis and Proliferation

**Fig 5:**
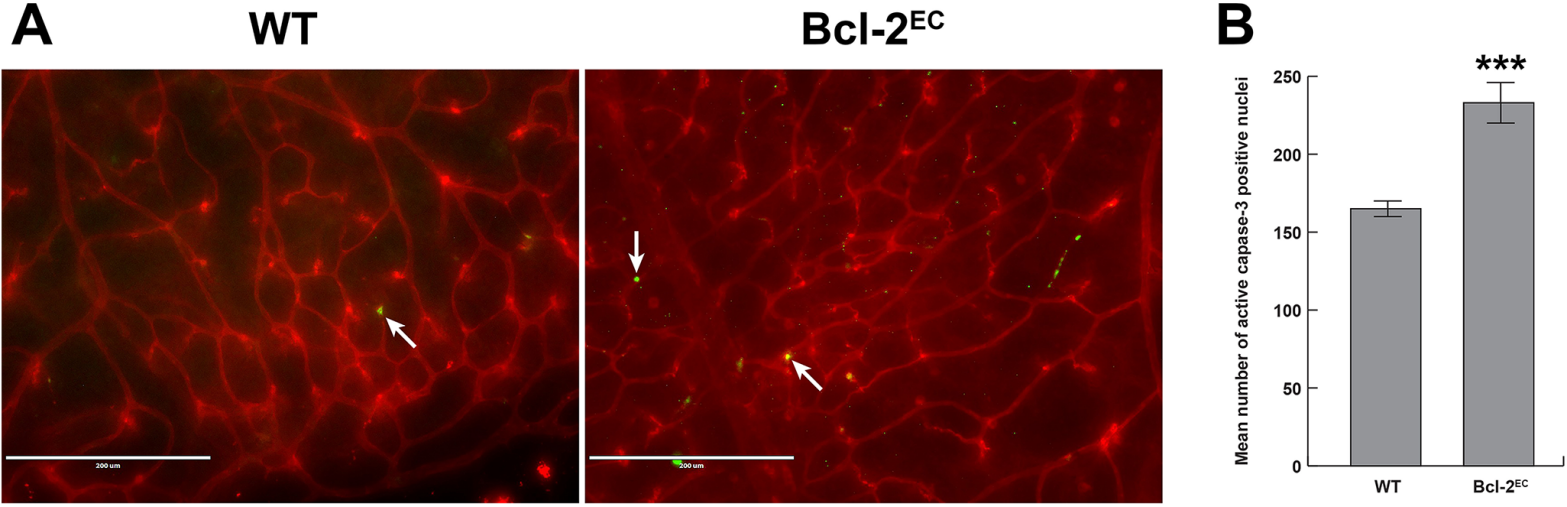
Increased apoptosis in retinas from Bcl-2^EC^ mice. In Panel A, retinas from P14 wild-type and Bcl-2^EC^ mice were wholemount stained with anti-cleaved caspase 3 and the intermediate layer imaged. The data is quantified in Panel B. The data in each bar are the mean number of cleaved caspase 3 positive cells counted in the vasculature of each retina. Arrows point to apoptotic cells. These experiments were repeated with at least 5 mice. ***P<0.001. The scale bar equals 200 μm.

**Fig 6:**
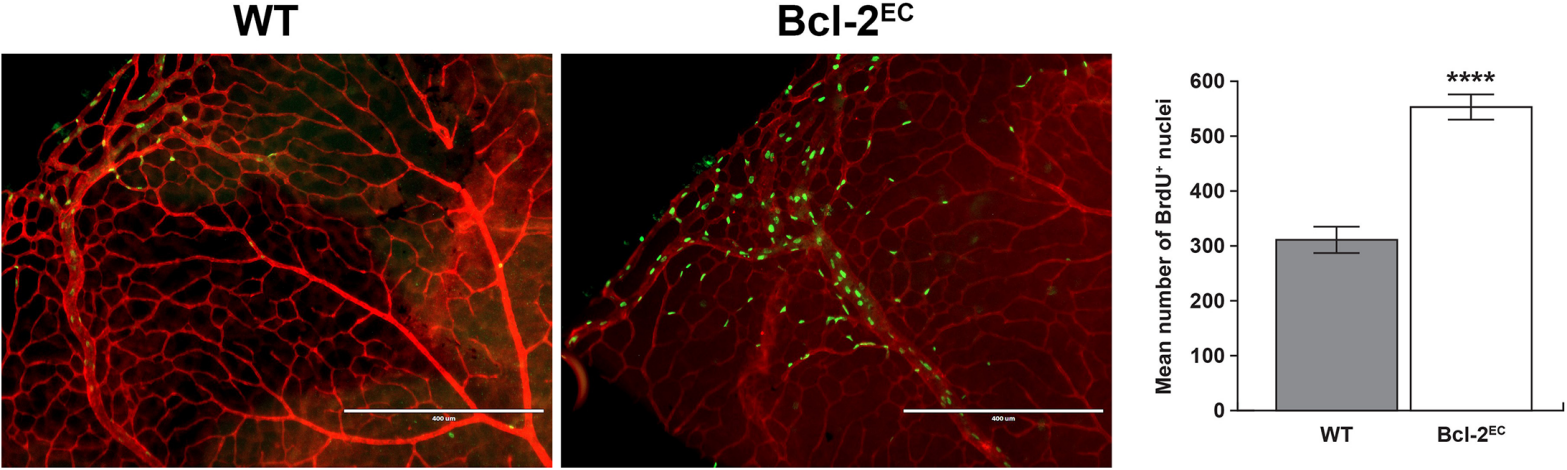
Increased proliferation in retinas from Bcl-2^EC^ mice. In Panel A, proliferating cells in P14 wild-type and Bcl-2^EC^ mouse retinas were labeled by BrdU and detected using an antibody to BrdU and co-stained with Isolectin-B4-FITC to visualize the vasculature. In Panel B, the data in each bar are the mean number of BrdU+ cells counted in each retina. These experiments were repeated with at least five mice of each genotype. Please note that the number of proliferating cells is higher in retinas from Bcl-2^EC^ mice compared to their wild-type counterparts (****P< 0.0001). Scale bar equals 400 μm.

Our previous studies in global Bcl–2 knockout mice demonstrated increased apoptosis and proliferation in several organs including the retina [14, 23]. Here, apoptosis was assessed by anti-active capase 3 and proliferation by BrdU staining of wholemount retinas from P14 mice. We observed modest levels of apoptosis in retinas from P14 wild-type mice (Fig 5), while retinas from P14 Bcl-2^EC^ mice demonstrated increased levels of apoptosis compared to their wild-type counterparts. As we had previously seen in global Bcl–2 knockout mice, the level of proliferation in retinas from Bcl-2^EC^ mice was significantly increased compared to their wild-type counterparts (Fig 6). Thus in the absence of Bcl–2, endothelial cell apoptosis and proliferation levels were significantly increased.

### Ischemia-Driven Retinal Neovascularization is Similar in Bcl-2^EC^ and Littermate Controls

**Fig 7:**
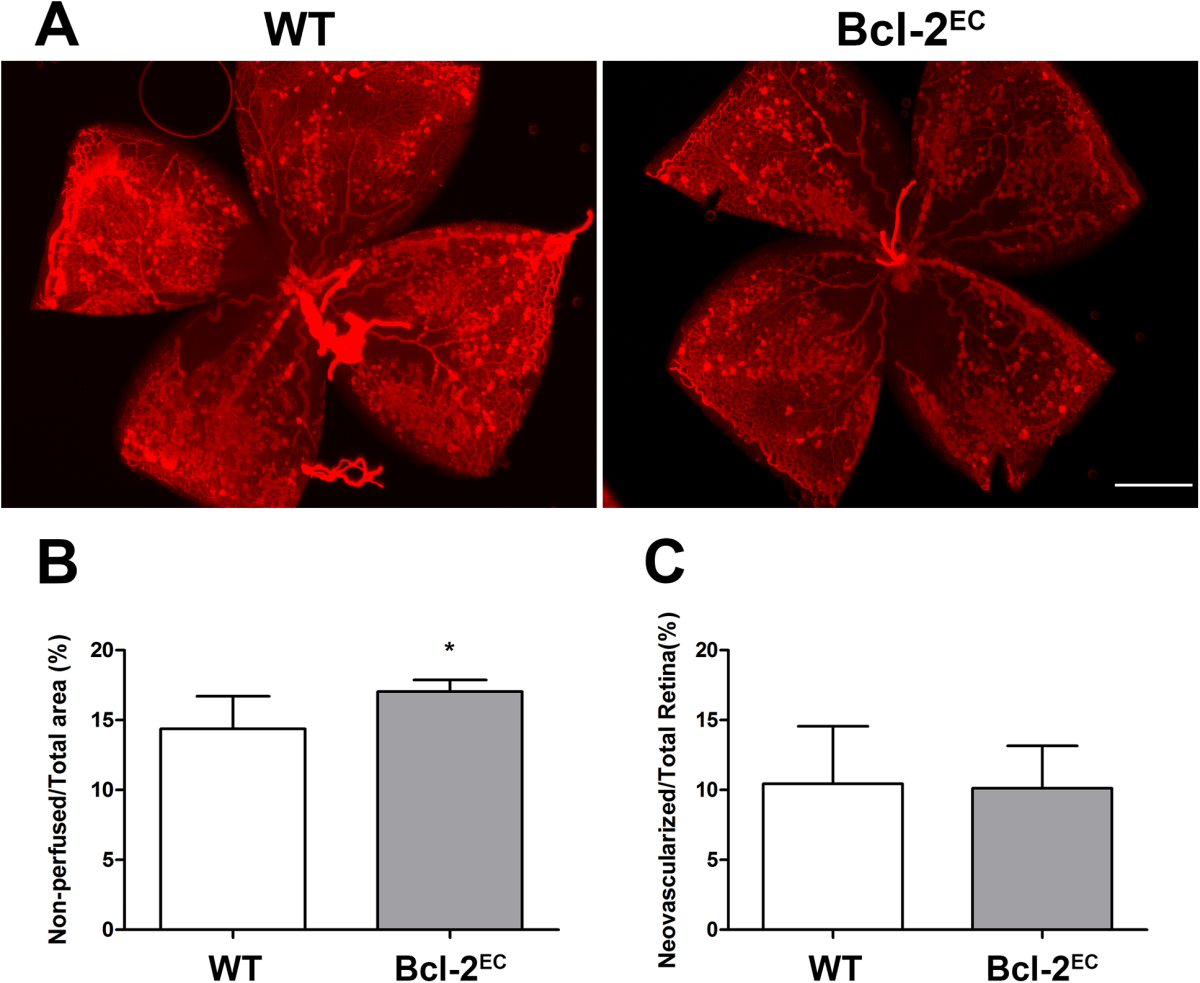
Lack of Bcl–2 expression in endothelial cells does not attenuate ischemia-driven retinal neovascularization. Quantitative assessment of neovascularization in P 17 mice exposed to a cycle of hyperoxia and room air (OIR). Retinas from Bcl-2^Flox/Flox^ (WT) and Bcl-2^EC^ littermates were wholemount stained with anti-collagen IV to visualize the vasculature (Panel A). The area of vessel obliteration (noted in yellow) relative to the whole retina was quantitated (Panel B; ***P<0.001). Panel C is a quantitative assessment of the neovascularization (P> 0.05). Experiments were repeated at least three times with 5 mice per group with similar results. Scale bar = 1000 μm.

Previous studies from our laboratory demonstrated that Bcl–2 expression was required for ischemia-driven retinal neovascularization that accompanies hyperoxia-mediated vessel obliteration during OIR [15]. To determine whether lack of Bcl–2 expression in endothelial cells was sufficient to prevent ischemia-driven retinal neovascularization following OIR, P7 mice were subjected to hyperoxia for 5 days and then returned to room air for 5 days. Both wild-type and Bcl-2^EC^ mice displayed hyperoxia-mediated retinal vessel obliteration and subsequent neovascularization (Fig 7). Although, Bcl-2^EC^ mice displayed a modest increased area of vessel loss following hyperoxia compared to their control littermates, they displayed similar levels of ischemia-driven retinal neovascularization. Therefore, unlike global Bcl–2 knockout mice, Bcl-2^EC^ mice were unable to suppress ischemia-driven retinal neovascularization.

### Lack of Bcl–2 Expression Attenuated Choroidal Neovascularization

**Fig 8:**
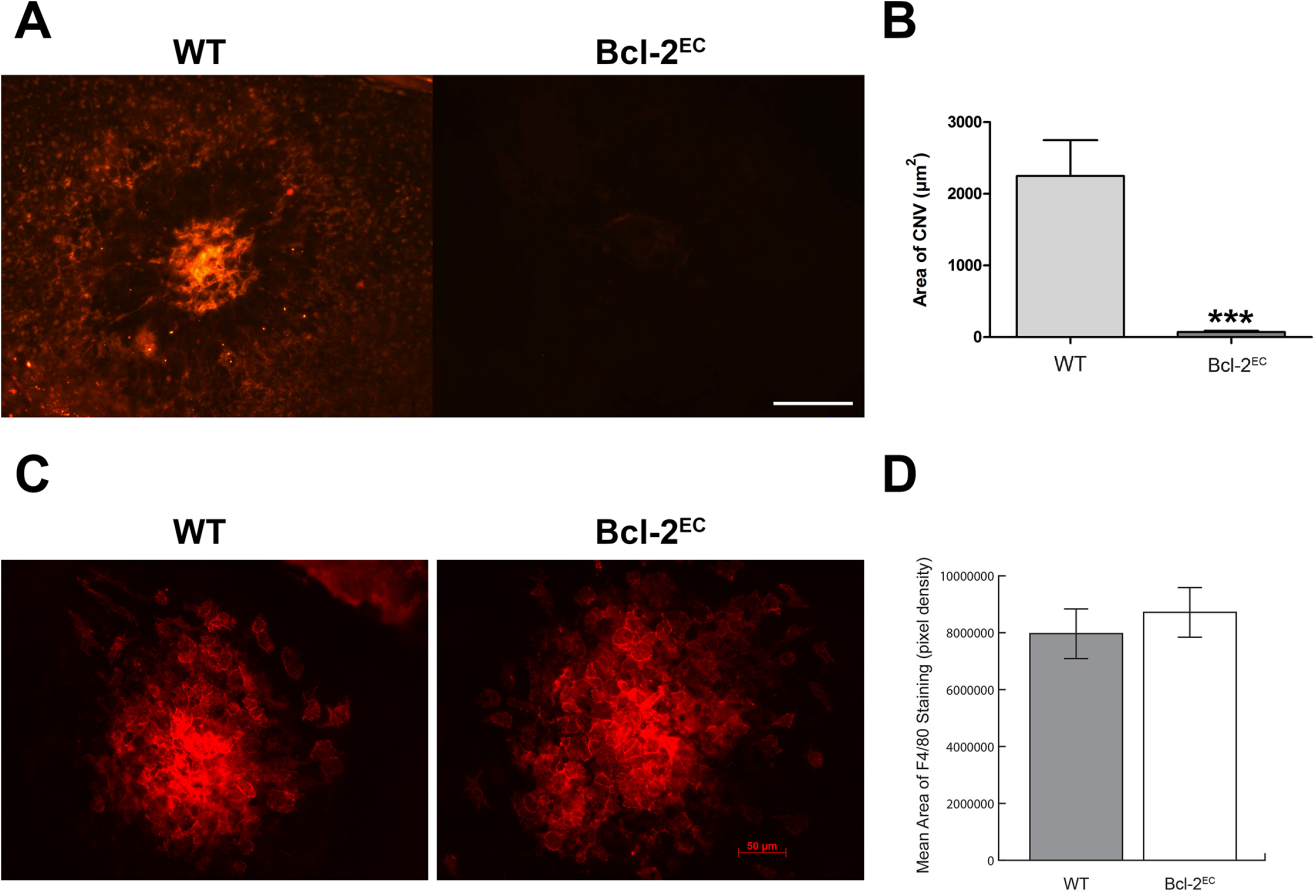
Lack of Bcl–2 expression in the endothelium attenuates choroidal neovascularization. Choroidal neovascularization was induced in six-week old female mice by laser photocoagulation-induced rupture of Bruch’s membrane. After 14 days (Panel A) the eyes were sectioned at the equator, and the anterior half/vitreous/retina removed. The remaining eye tissue was stained with anti-ICAM–2 and the area of neovascularization quantitated (***P< 0.001). Scale bar = 100 μm. In Panels C and D, the choroid was harvested on Day 3 post-laser and F4/80 staining performed (P>0.05). The intensity of staining was quantitated by Image J. Please note choroidal neovascularization is inhibited in the absence of Bcl–2 while recruitment of macrophages to site of lesion was not affected. These experiments were repeated with at least 8 mice of each genotype.

The other major circulation that nourishes the outer retina is choriocapillaris, whose dysfunction contributes to the pathogenesis of age-related macular degeneration and CNV. Although retinal neovascularization and CNV are thought to share molecular signals, the role hypoxia plays during CNV remains questionable [24]. In addition choroidal endothelial cells are fenestrated, unlike retinal endothelial cells [25]. Perhaps this may be why our previous studies have shown these processes are separable [14]. Here we determined whether endothelial Bcl–2 expression influenced choroidal neovascularization. Female 6-week old mice had CNV induced by laser photocoagulation-induced rupture of Bruch’s membrane on Day 0. The eyes were harvested 2 weeks later and the area of CNV was determined by ICAM–2 staining. Bcl-2^EC^ mice demonstrated significantly less CNV than their wild-type counterparts (Fig 8A and 8B). To determine whether the decrease in CNV in Bcl-2^EC^ mice was attributed to a decline in macrophage infiltration, choroid/RPE samples were harvested at Day 3 post-laser and stained with F4/80. Fig 8C demonstrates a similar amount of F4/80 staining in choroid from wild-type and Bcl-2^EC^ mice. Although Bcl–2 expression in the endothelium did not impact the recruitment of macrophages to the site of laser photocoagulation, its expression is essential for proangiogenic activity and CNV.

## Discussion

Regulation of angiogenesis is essential during vascular development and neovascularization. Although the role Bcl–2 plays during angiogenesis is beginning to emerge, its contribution in the endothelium during angiogenesis is not well understood. Generation of mice lacking Bcl–2 expression in the endothelium allowed us to dissect the role Bcl–2 plays during formation of the retinal superficial vascular layer, artery formation, vascular pruning and remodeling, and pathological neovascularization. During the first week of life the mouse retinal superficial vascular layer is formed, followed by formation of the deep and intermediate vascular plexus. Here we examined remodeling of unwanted retinal vessels that occurs following completion of the vascular plexus [26]. Although mice lacking endothelial Bcl–2 expression had fewer retinal endothelial cells at 3 weeks of age compared to wild-type mice, they still demonstrated loss of retinal endothelial cells during remodeling (between 3 and 6 weeks of age) albeit at a slightly exaggerated rate compared to wild-type mice. Pericyte numbers only decreased between 3 and 6 weeks in retinas from wild-type mice. Perhaps pericyte numbers are maintained in Bcl-2^EC^ mice in an attempt to preserve retinal vascular integrity. Thus, even though Bcl-2^EC^ mice have decreased numbers of retinal endothelial cells, loss of endothelial cells during retinal vascular pruning and remodeling is still observed.

The characteristics of the apoptotic cells were first described over 40 years ago by Kerr et al [27] and was followed by steadily increasing study of this area. Cell lineage studies in the nematode *Ceaenorhabditis elegans (C*. *elegans)* set the stage for our understanding of the evolutionary conservation and genetic regulation of programmed cell death [28, 29]. These studies demonstrated that of the 1090 somatic cells generated during *C*. *elegans* development, 131 cells underwent apoptosis resulting in a ~12% cell loss [28]. Three genes were found to be important in regulating apoptosis during this process including *ced–9*, which prevented cell loss [30, 31] and possesses sequence and functional similarity to Bcl–2. Here we observed that loss of retinal endothelial cells during remodeling has similarities to *C*. *elegans* developmental cell loss. We observed approximately an 11% loss of retinal endothelial cells in wild-type mice between 3 and 6 weeks of age. Like *C*. *elegans*, mice lacking Bcl–2 in endothelial cells demonstrated an exaggerated loss of retinal endothelial cells during pruning and remodeling. Thus, developmental remodeling or the regulated “pruning” of unneeded cells by apoptosis may have similarities across multiple developmental systems.

Branching of the ophthalmic artery supplies the central retinal artery and choroid. The central retinal artery enters the eye at the level of the optic nerve head sending branches over the internal surface of the retina. In humans the artery divides within the optic nerve forming two major trunks which will each branch again to supply the retina. We normally observed 5–6 retinal arteries supplying the quadrants of the mouse retina, with retinal veins alternating. Primary branches off these arteries were normally observed at a distance greater than 600 μm from the optic nerve head. In Bcl-2^EC^ mice we typically observed 4–5 retinal arteries and correspondingly fewer veins. Primary branching off the retinal artery occurred prematurely in these mice. We also saw decreased secondary branches off the retinal arteries in Bcl-2^EC^ mice consistent with decreased retinal vascularity. These data confirm our previous studies in global Bcl–2 deficient mice which demonstrated decreased numbers of retinal and renal arteries [15] and suggest an important role for Bcl–2 in the endothelium during arteriogenesis.

Premature primary branching in the absence of Bcl–2 is not unique to the retinal vasculature. Our previous studies in the kidney demonstrated aberrant ureteric bud branching in the absence of Bcl–2 [32]. Global Bcl–2 deficient mice demonstrated premature primary branching of the ureteric bud as well as decreased numbers of ureteric branch points and branch tips. These changes contributed to decreased nephron number and renal mass which was partially restored by reexpression of Bcl–2 in the ureteric bud in global Bcl–2 deficient mice [6]. Culture of Bcl–2 deficient ureteric bud cells, like Bcl–2 deficient retinal endothelial cells, demonstrated aberrant cell adhesion, migration and extracellular matrix production which culminated in an inability of these cells to undergo branching morphogenesis in Matrigel [7, 32]. Thus, our data suggest shared branching morphogenesis pathways of the epithelium and endothelium, positioning Bcl–2 as a key regulator of cell adhesive mechanisms important during this process [33].

Several laboratories including ours have shown that Bcl–2 expression plays a critical role during angiogenesis [7, 15, 34–37]. Our previous studies demonstrated that lack of Bcl–2 expression attenuated ischemia-driven retinal neovascularization following OIR even though hyperoxia-mediated retinal vessel obliteration was still observed [15]. Here we determined whether lack of Bcl–2 expression in endothelial cells was sufficient to prevent retinal neovascularization accompanying OIR. Bcl-2^EC^ mice demonstrated increased hyperoxia-mediated retinal vessel obliteration and similar levels of ischemia-drive retinal neovascularization compared to controls. Unfortunately, the processes involved in choroidal neovascularization are less defined [24]. In contrast to retinal neovascularization observed during OIR, Bcl-2^EC^ mice demonstrated attenuation of laser-induced CNV, even though macrophage recruitment to the site of laser photocoagulation was not impacted. As we have previously seen, these data are consistent with retinal and choroidal neovascularization being regulated in different manners [14]. Thus, Bcl–2 may provide a suitable target for treatment of CNV associated with AMD. It remains to be seen whether endothelial cell fenestration, hypoxia, or stage of development plays a role in the differential ability of these mice to undergo neovascularization in the absence of Bcl–2. However, it is tempting to speculate that vessel origin may have an influence since both the choroid and retinal arteries derive from the ophthalmic artery. In the absence of endothelial Bcl–2 expression, aberrant vascularization was observed both in number of retinal arteries and neovascularization with CNV. Alternatively, lack of Bcl–2 expression may result in a defect in endothelial cell function such as migration, adhesion or proliferation all of which we have observed in Bcl–2 -/- vascular cells *in vitro* [7, 8]. Many if not all of these processes are developmentally regulated which may be why we observed a difference of the ability of Bcl-2^EC^ to neovascularize in the OIR and CNV models. Thus, gaining a better understanding of the role Bcl–2 plays in specific vascular cells will give us new insight into novel ways to intervene, to either promote desirable or prevent unwanted vascularization.
